# Microbial Transglutaminase Is Immunogenic and Potentially Pathogenic in Pediatric Celiac Disease

**DOI:** 10.3389/fped.2018.00389

**Published:** 2018-12-11

**Authors:** Matthias Torsten, Lerner Aaron

**Affiliations:** ^1^AESKU. KIPP Institute, Wendelsheim, Germany; ^2^B. Rappaport School of Medicine, Technion-Israel Institute of Technology, Haifa, Israel

**Keywords:** celiac disease, microbiome, food additive, gluten, cross linking, microbial transglutaminase

## Abstract

The enzyme microbial transglutaminase is heavily used in the food processing industries to ameliorate food qualities and elongate the products' shelf life. As a protein's glue, it cross-links gliadin peptides, creating neo-complexes that are immunogenic and potentially pathogenic to celiac disease communities. Even lacking sequence identity, it imitates functionally the endogenous tissue transglutaminase, known to be the autoantigen of celiac disease and representing an undisputable key player in celiac disease initiation and progress. The present review expend on the enzyme characteristics, exogenous intestinal sources, its cross-linking avidity to gluten or gliadin, turning naïve protein to immunogenic ones. Several observation on microbial transglutaminase cross linked complexes immunogenicity in celiac patients are reviewed and its pathogenicity is summarized. Warnings on its potential risks for the gluten dependent conditions are highlighted. When substantiated, it might represent a new environmental factor of celiac disease genesis. It is hoped that the presented knowledge will encourage further research to explore the mechanism and the pathogenic pathways taken by the gliadin cross linked enzyme in driving celiac disease.

## Introduction

Celiac disease (CD) is an autoimmune disease, thus dependent on genetic background and on environmental factors. The list of the environmental components that might impact CD initiation or development is continuously expanding, but the precipitating events causing CD remain enigmatic. Except for the prolamins, none of them reached cause and effect relationship. The introduction is divided to 4 sub-heading in order to set the stage for microbial transglutaminase (mTg)-CD cross-talks.

### Environmental Factors Associated With Celiac Disease

A plethora of environmental components were suggested to influence CD, spanning infections, food, drugs, vaccination, toxins and metals, abdominal or gynecological surgery, level of hygiene, socio-economic status, life style stress and processed food additives (1–5). Microbes like *Helicobacter pylori, Campylobacter jejuni*, pneumococcus, bacteroides species and tuberculosis were associated. In the virus's domain: CMV, HBV, HCV, Rotavirus, Adenovirus, Reovirus, and enteroviruses were implicated. To complicate the topic, same bacteria and viruses might play a protective role in CD (4). Pharmacological factors are early antibiotic therapy and proton pump inhibitors. Influenza and HPV vaccines, Heavy metals, Aflatoxin, smoking, alcohol, Cesarean section and abdominal surgery, Western higher hygiene and higher socioeconomic level, stressful life and Westernization of the diet, microbiome dysbalance characterized by an abundance of Proteobacteria and a decrease in Lactobacillus were associated with CD development or increased incidence (6, 7). Most recently the processed food additive were suggested to influence the intestinal microbiome and to increase intestinal permeability, thus contributing to luminal eco-events that drive autoimmunity (8). One of them is the mTg, the topic of the present review. In 2015, the hypothesis that mTg might play a role in CD genesis was forwarded and since then more studies were conducted to substantiate this hypothesis (9).

### MTg: Characteristics, Functions, Food Applications

Prokaryotic mTg is a part of the global transglutaminases family (10), isolated and characterized from multiple bacterial strains, *Streptomyces mobaraense* being the first one (11, 12). The *S. mobaraense* mTg harbors 331 amino acids, its molecular weight is 37.9 kDa. Notably, list of additional microbes that secrete the enzyme and their enzymatic yield capacities is continuously expanding (13), a recent one is a novel Transglutaminase from *Streptococcus suis* (14).

Being a member of the Tg family, mTg catalyzes the formation of an isopeptide bond, cross-linking an amine group (containing the acyl acceptor lysine) and the acyl group (containing the acyl donor glutamine). As gluten is abundant in glutamine (~30%) and contains lysine (<2%) (15), representing an acyl donor and acceptor, respectively, it is an ideal substrate for post-translational modification of gluten, by transamidation, or deamidation. Based on its peptide modifying capacity it imitates functionally the endogenous tTg, the ultimate autoantigen in CD (9, 13, 16, 17). It should be emphasized that bonds formed by the mTg resist proteolytic degradation, it exhibits a higher reaction rate, substrate specificity, higher transamidation/ deamidation ratio resulting in improved cross-linking capacity. Due to its broad enzymatic activity, it is heavily used by the food processing industries (8, 9, 12, 18–23). In fact, the enzyme is consumed by most of the processed food industries, spanning the meat, dairy, sea food and fish, surimi, casein and gelatin, myosin and actin, confection, and convenience ones and many more (8, 9, 12, 18–22). The net % increase per year of enzyme usage in the processed food industries is estimated to 21.9%, mTg being a major one (8). In the food processed industries, mTg improves gelation and changes emulsification, foaming, viscosity and water-holding capacity. It is considered as the “glue of proteins” and polymerization agent, thus improving food palatability, texture and life time on the supermarkets' shelves.

Zooming on CD and the increased affinity of the mTg to gluten, the enzyme in increasingly used in the bakery industries (8, 9), where it lowers the products calories, improves texture, elasticity and dough characteristics (9, 12, 24).

Summarizing the literature, an estimated daily intake of mTg used in the processed food products can range up to 15 mg where every kg of mTg processed product contains around 50–100 mg of mTg (18, 25, 26). Finally, a direct positive correlation is found between the increased annual usage of industrial enzymes added to processed bakery products and the increase in CD frequency, in the last four decades (7, 13). It should be stressed that we are dealing with an associative correlation and no causality was determined.

### MTg Is Structurally Different but Functionally Imitates the tTg

In contrast to endogenous human tTg, the microbial one is a calcium and nucleotide independent enzyme. It consists of a single domain, compared to the four domains of the tTg and has around half the molecular weight. MTg operates at a larger range of pH, buffers, and temperatures and has a much larger list of substrates. So, in opposition to the tTg, mTg less substrate specific. Those features are advantageous for many industrial applications and it is clear why the industries preferred mTg over its member, tTg. MTg lacks sequence homology to tTg, but, due to their active site performances, mTg has substantial functional similarity (9, 13, 18). Not surprisingly, the two enzymes can transamidate or deamidate proteins, based on the enzymatic reaction's conditions (23).

### The Luminal Intestinal Sources of mTg

MTg can originate from intra luminal sources and from external sources. The luminal enteric sources come from the gut microbiome. Our lumen is over-crowded by bacteria but also by archaea, viruses and protozoa, reaching roughly 10^14−16^, and the ratio of microbes/ host cells ratio averages 1:1. Its dimensions, composition, diversity, mobile products and activities have led to its description as a “superorganism” (27). A component of the microbial Metabolome represented as a mobilome is the secreted mTg. It appears that by using sequence search programs, hundreds to thousands of mTgs-encoding bacteria can be detected, the majority belong to the Firmicutes phylum (13, 16). As a survival factor mTgs are secreted by the microbes in order to survive their overpopulated luminal compartment. When intestinal fluid was analyzed, the fluid was positive for transglutaminase activity, however, the authors did not distinguish between the endogenous tTg and the bacterial one, but to our knowledge, no tTg activity was reported in the duodenal lumen, so far, in contrast to the intestinal mucosa and the lamina propria (28).

The extra intestinal sources of mTg are much wider. Mtg as a processed food additive was extensively discussed above (8, 9, 12, 13, 16, 18–22). Belonging to the prokaryotes, the probiotics represent a reservoir of mTg, representing an active cargo that affects luminal events. It is well-known that microbes can transfer virulent factors to their mates by horizontal gene transfer (29). Major concern on the transfer of virulent genes, like antibiotic resistant genes, via the nutrients and food chain exists and many of those deleterious genes are carried by probiotics (30–32). The virulent genes were described to end up in the human gastrointestinal tract microbiota (33). The factors causing the transformation from benign inhabitant of the gut microbiome or ingested probiotics to virulent pathogen are not clear, but a combination of horizontal gene exchange of virulence factors and differential transcription of endogenous genes are clearly involved (34).

An additional enzymatic cargo of mTg delivered to the enteric lumen comes from the pathobionts. In addition to their capacity to post-translate and modify protein and breach tight-junction integrity (8, 13, 16) they add pathogenic burden, represented by the mTg, as was recently described (14). The environmental pathogenic microbes can exchange mobile elements with the luminal inhabitants. Surface water, sewage treatment plant effluents, soils, animal wastes, contaminated biofilms and urban rivers are increasingly reported, thus threatening global human health (35–37). Not surprising, commonly consumed plants and vegetables contain transglutaminase. Even if their sequence homology to mTg is not high, they are capably to cross-link peptides, including gluten. They were even implicated as a possible player in CD pathogenesis, starting the process in the intestinal lumen (38). MTg carrying examples are apple, soybean, bean sprouts, fodder beet, rosemary leaves, Jerusalem artichoke, spinach leaves and green peas, routinely consumed/ingested plants, fruits and vegetables, all have transglutaminase activities (38). Polyols, heavily used in the processed food industry in protein-based coating, biofilm formation, gelatination of products and bio-packaging, were shown to improve thermal stability and half-life of the mTg, thus potentiating the enzyme cross linking capacity (39).

A totally new topic of potential Tg delivery to the gut lumen is coming from the microbiota yeast's domain. Significantly higher fecal counts of candida and saccharomyces species were identified in CD patients (40) and CD patients have higher frequency and levels of anti-*Saccharomyces cerevisiae* antibodies (41). Both, *Candida albicans* and *Saccharomyces cerevisiae* synthesize the Tg enzyme (42, 43) to the point that *Candida albicans* was suggested to trigger CD (44, 45).

Summarizing the present paragraph, a plethora of extra intestinal and luminal sources of mTg exist that potentially can interact with gluten containing compounds. This enzymatic cargo can cross link those glutamine rich peptides, resulting in post-translated, modified immunogenic epitopes, potentially driving CD autoimmunogenesis (9, 13, 16). After setting the introductory stage, the following part will expend on the mTg cross linked gliadin complex immunogenicity and potential pathogenicity in CD.

## Gliadin Docked mTg Complexes are Immunogenic in Pediatric CD

When mTg transamidates gliadin peptides a neo cross-linked complex is created, where the physico-chemical, electrical and three dimensional features are changed (46). This is a typical enzymatic post-translational modification of gliadin, resulting in a non-naïve, non-tolerogenic and potentially immunogenic protein (13, 16). To investigate its immunogenicity, CD associated antibodies, including the anti-neo-epitope mTg were investigated, back to back, in a pediatric CD population, compared to controls. The neo-epitope mTg IgG had a sensitivity of 94.9% and specificity of 93.9% and reflected the patient's intestinal pathology (46). Performing competition assays between the isolated antigens (gliadin, tTg and deamidated gliadin peptide) and the gliadin tTg/mTg neo complexes, mTg and tTg-neo epitopes display comparable immunopotent epitopes (46).

After describing the mTg-gliadin complex antigenicity in CD, the stage is ready to evaluate mTg potential pathogenicity in driving CD.

## Pathogenic Aspects of mTg

Several aspects of the mTg hint at its potential pathogenicity and suggest involvement in CD initiation/progression. Sharing functional aspects with the autoantigen and the mucosal step limiting phase of the tTg enzyme in CD development puts mTg as a primary candidate as a partner for CD development. Following are several observations that support mTg involvement in the CD autoimmunogenesis.

### MTg Suppresses Intestinal Luminal Protective Barriers

The human intestine possess multiple protective mechanisms to keep the microbes at bay. Several of those mechanisms are contradicted by the mTg, when bugs are fighting to survive inside us:

- The highly physiological important isopeptide bonds created by the mTg are resistant to any known human enzyme, thus escaping the enzymatic hydrolysis, reducing or chaotrophic agents and detergents. Those mTg formed protective structures improve the microorganism's survival in the enteric hostile compartment. Even bile acids, antimicrobial peptides and immunoglobulins are ineffective facing those structures (13, 47, 48).
- mTg was found to suppress human immunity by its anti-phagocytic property, thus suppressing a major immune protective system (14, 49, 50).

### MTg Effects on Epithelial Gliadin Uptake and Transportation

- MTg may enhances gliadin uptake through human intestinal cell-line, as was demonstrated for tTg. TTg was shown to facilitate apical-basal passage of gliadin peptides, cooperating with the apical transferrin receptor and secretory IgA (9, 13, 51). Imitating tTg functions, mTg potentially can facilitate this epithelial gliadin uptake pathway, thus enhancing CD.
- MTg and gliadin share comparable trans-enterocytic transport pathway. A major indispensable step in the pathophysiology of CD is the transcytosis of gliadin peptide to the sub-epithelial space to be deamidated\ transamidated by the tTg. It appears that mTg and gliadin peptides use this port of entry and pathway. Most Lately, Stricker et al. (52) placed tagged mTg and gliadin to the enterocyte upper membrane of CD and control human intestinal specimen or human cell line. A simultaneous uptake of mTg and gliadin into the endoplasmic reticulum of the RACE-cells and co-localization of the mTg and gliadin at the basolateral membrane and the lamina propria of CD duodenal samples were detected. If their results are substantiated it might reinforce the potential pathogenic role of the exogenous mTg or mTg-gliadin complex in CD evolution, when the complexes are exposed to the local immune systems. In this case, one can envision that the sub-epithelial deposits of tTg-gliadin, described as an early histological finding in CD intestinal samples, contain mTg-gliadin complexes.

### MTg can Potentially Enhance Gliadin Induced Intestinal Permeability

Several mechanisms can explain how mTg functions and products can increase enteric permeability:

- Protecting intestinal dysbiota and pathobionts, the mTg can enhance their luminal survival. Intestinal infections are important breachers of tight junction functional integrity (53).
- Gliadin is an ideal substrate for mTg cross-linking. Being a major disruptor of intestinal permeability (54), its cross-linked complexes might further augment gut permeability.
- Some tight junction proteins and cytoskeleton elements like actin, e-cadherin or adherens junctions can be modified by Tgs (55, 56). By cross-linking those proteins the mTg can disrupt tight junction functionality (9).

### Additional Facts Related to Potential mTg Pathogenicity in CD

In order that the industrial processed food additive will impact human health, it has to be present in the consumed products. Notably, when 60 meat and meat products from the supermarket shelves were checked by two sensitive (around 25 mg pure enzyme in 1 kg of product) analytical methods, many contained the enzyme mTg (57). A literature survey disclosed that around 50–100 mg of mTg is used to process 1 kg of food product and the average intake of mTg can amount to 15 mg per day (12, 18, 25, 26). Although not causative, epidemiological data show an associative correlation between the surges of CD incidence and the consumption of enzymes in the bakeries, mTg being a major one (8, 9). Finally, wheat or gluten containing products enzymatically treated by mTg were shown to be immunogenic, inducing antibodies when consumed by humans (9, 46, 58–67).

## Academical and Authoritative Warnings on the Potential Harmful Functions of mTg Usage by the Processed Food Industries

The mTg is not labeled, since it is considered as a processing aid, thus escaping the definition of a food additive. Due to the potential detrimental public health aspects, several scientists and organizations issue warnings, trying to increase the awareness of the regulatory authorities, academical communities and the general public on the subject. Following are some citations: “The usage of transglutaminase as a food additive is permitted in some countries. However, its utilization has to be declared to ensure transparency for consumers” Kaufmann et al. (57), “Therefore mTg can enhance the immunogenicity of gluten and should not be used in food products intended for consumption by CD patients” Dekking et al. (66). Not surprisingly, the worries and warnings on mTg nutritional industrial usage safety appear in numerous publications (26, 38, 57, 62, 66–68). More so, at least, in Switzerland and Germany, public warning were issued concerning mTg food safety, recommending labeling of the enzyme (69)^1^. The warnings mentioned the risk of mTg consumption by CD patients, “Suitable labeling of foods produced using mTg would enable these patients to avoid the uncertainties that the scientific community has yet to clarify”^1^.

## Conclusions

**Figure 1:**
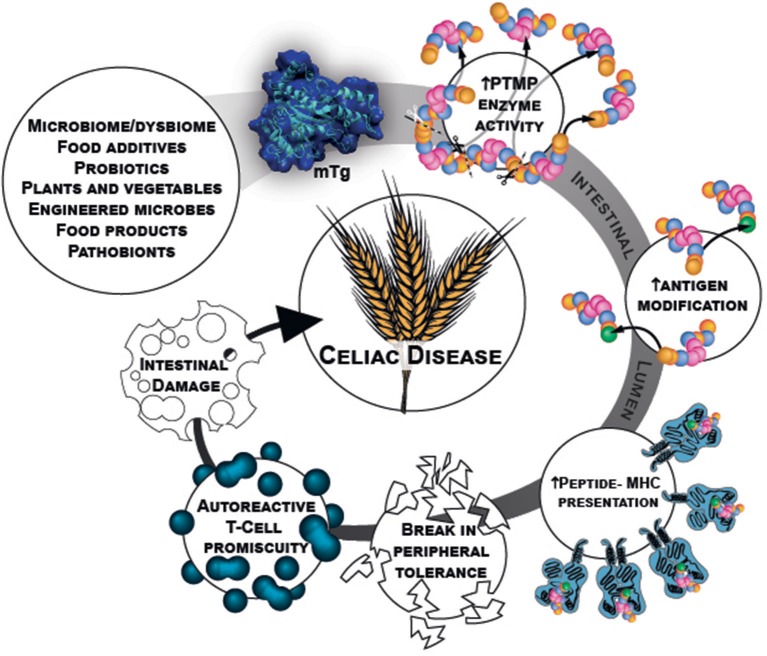
mTg sources and chain of events that potentially initiate gluten break of tolerance resulting in CD.

Microbial transglutaminase is a food additive, heavily used in a plethora of processed food industries. It is unlabeled and hidden from the public knowledge. Being functionally similar to the tTg, it can post-translate and modify gliadin peptides by cross-linking them, thus, inducing loss of tolerance. Figure 1 describe schematically the chain of events starting from the mTg sources ending in CD development.

There are published warnings, alarming the public on the potential danger of using or consuming this enzyme. Recent publications found mTg to be immunogenic in CD patients and its pathogenicity is continuously unraveled. The logical theoretical basis for the mTg to be a new environmental factor in CD induction exist, however, causality should further be explored.

## Author Contributions

MT designed, overviewed, searched, and analyzed the literature and edited the manuscript. LA designed and wrote the manuscript.

### Conflict of Interest Statement

MT is the head of the Aesku. KIPP institute. The remaining author declares that the research was conducted in the absence of any commercial or financial relationships that could be construed as a potential conflict of interest.

## Footnotes

^1^http://www.bfr.bund.de/cm/343/transglutaminase-in-fleischerzeugnissen.pdf

## Abbreviations

CD: celiac disease
mTg: microbial transglutaminase
tTg: tissue transglutaminase.
